# Synthesis of polymeric 2D-graphitic carbon nitride (g-C_3_N_4_) nanosheets for sustainable photodegradation of organic pollutants

**DOI:** 10.1016/j.heliyon.2024.e33354

**Published:** 2024-06-25

**Authors:** Afroja Banu, Biswajit Sinha, Suranjan Sikdar

**Affiliations:** a Department of Chemistry, University of North Bengal, Darjeeling, 734014, India; b Department of Chemistry, Ghani Khan Choudhury Institute of Engineering and Technology (GKCIET), Narayanpur, Malda, 732141, India

**Keywords:** Graphitic carbon nitride, Thermal condensation, Organic dye pollutants, *p*-Nitrophenol, Photocatalyst, Urea

## Abstract

A superficial, one step thermal polycondensation method has been employed for the manifestation of graphene like graphitic carbon nitride (g-C_3_N_4_) catalyst. The as synthesized g-C_3_N_4_ was well characterized by SEM and EDAX analysis, XRD, ATR-IR, FTIR, Fluorescence spectroscopy, Raman spectroscopy and UV–Visible spectroscopy which provide structural, morphological assemblage relating to the structure of g-C_3_N_4_. The g-C_3_N_4_ showed that an outstanding photochemical stability, morphology, conductive carbon framework and superior photocatalytic activity. The band gap value of g-C_3_N_4_ is 2.34 eV determined using Tauc plot. Due to low band gap (2.33 eV) and unique morphology which provides high separation and migration ability of the photogenerated charges, the g-C_3_N_4_ shows enhanced photocatalytic activity for the removal of many organic dyes such as Rhodamine B (RhB), Crystal Violet (CV), Methylene Blue (MB), Methyl Orange (MO), Naphthol Orange (NO) and a phenol derivative, *p*-Nitrophenol (p-NP). Among them, RhB dye was degraded almost 81 % at 90 min under sunlight irradiation in presence g-C_3_N_4_ while other dyes and p-NP was degraded at lower rate. From the experimental data, it was found that MO and p-NP degradation rate was least. The rate constant for degradation of Rh B is 1.1 × 10^−2^ min^−1^. Therefore, g-C_3_N_4_ can be used as an efficient photocatalyst for waste water treatment by the removal of such organic pollutants.

## 1 Introduction

Rapid urbanization, off-the-wall industrialization, and economic growth with intensifying abuse of available resources have outcome as a threat to the mankind and environment making it unsustainable and deficit in pure water supplies. Waste water treatment is becoming the new challenge to save the environment from the toxic pollutants generated from various sources like industries and agricultural fields with the help of nanomaterials. Removal of this toxic, non-biodegradable, haunting organic pollutants is unenviable, knotty and costly [1]. Organic pollutants such as dye pollutant and some phenol derivatives present in wastewater are toxic and harmful to human health as well as the environment [[2], [3], [4], [5]]. Photocatalysis is the most hard-hitting process for waste water treatment among several reported advanced oxidation processes (AOPs) like Fenton reaction, Ozonation, and others [6]. The mechanistic way of photocatalysis comes to the production of strong oxidants (h+) in the valence band (VB) and reductant (e^_^) in the conduction band (CB) via absorption of visible light by a semiconductor [7]. The interfacial charge transfer produces several reactive oxygen species (ROS) such as hydroxyl radicals, peroxide, and superoxide radicals which causes the effective degradation of organic pollutants [[8], [9], [10]]. Efficiency of a photocatalyst relies on suitable band gap and optimized charge carrier separation under visible light irradiation [11]. Some popular metal oxide photocatalyst such as TiO_2_, SnO_2_ and ZnO possesses large band gap, large surface area and vast electron capturing sites due to several defects originates from oxygen non-stoichiometry [[12], [13], [14]]. These traditional metal oxides can function as a photocatalyst only under ultraviolet light [15]. Because of the wide band gap, they can absorb lesser solar radiation which limited the practical applicability.

**Scheme 1:**
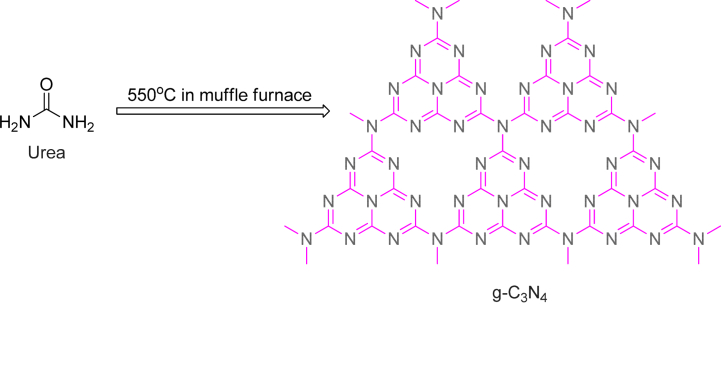
Synthesis of two dimensional layered g-C_3_N_4_.

To overcome the limitation, we have chosen graphitic carbon nitride (g-C_3_N_4_), one of the carbon nitride allotropes, a metal free photocatalyst consisting of tri-*s*-triazine subunits linked by planar tertiary amino groups in layer form. In the recent research works, g-C_3_N_4_ has mostly been reported to possess as block-like structure in which several nano-layers are stacked. This stacked block structure hardly affects the electron transfer rate [16]. g-C_3_N_4_ (2.34eV) has attracted considerable interest because of its large specific surface area, high stability, and optical response in the visible region [17]. The layered g-C_3_N_4_ material draw immense attention in the field of science due to its outstanding photochemical stability, affordable and low budget synthesis, precursor availability and moreover its non-toxic nature [[18], [19], [20]]. Despite the many advantages of g-C_3_N_4_, the rapid recombination of electron-holes caused by π-conjugation effect severely limits its development; in particular, layer stacking formed by strong hydrogen bonding between layers leads to a small specific surface area [21]. g-C_3_N_4_ has diverse applications in exploitation of biosensors [22], diagnostic imaging [23], therapeutic applications [24], photocatalysis [25], heterogenous photo-Fenton catalyst [26] for wastewater treatment and drug delivery [27] because of the large surface area, better electron movement, inherent medium band gap (2.7 eV) and distinctly defined chemical structure [28]. These properties would accelerate the photoinduced charge transfer and foreclose the backward reaction by disentangling the active sites for enhanced photocatalytic action [[29], [30], [31], [32]]. These commendable properties and applications brought about the potency of g-C_3_N_4_ to be a superior visible light responsive photocatalyst. In this work, we use bottom-up strategy to synthesis g-C_3_N_4_ via one step thermal polycondensation method using urea as the precursor at high temperature shown in Scheme 1 [33]. It was reported from several studies that the morphology of the g-C_3_N_4_ plays a dominant role in emphasizing the photocatalytic performance. Another factor in determining the photocatalytic performance is the precursor that has been used to prepare the g-C_3_N_4_. It was reported that the photocatalytic performance of g-C_3_N_4_ synthesized by using urea as precursor is better than others precursor derived g-C_3_N_4_ in case of Methylene Blue dye degradation [34].

The synthesized g-C_3_N_4_ photocatalyst was tested over various toxic dyes such as Methylene Blue (MB), Rhodamine B (RhB), Crystal Violet (CV), Methyl Orange (MO), Naphthol Orange (NO), *p*-Nitrophenol (p-NP) and the degradation outcome is surprising. We are amused by the photocatalytic performance of the urea derived g-C_3_N_4_ photocatalyst against these organic dyes and p-NP. Pure and as synthesized g-C_3_N_4_ degraded almost all the dyes which were chosen to perform in the photocatalytic experiment and g-C_3_N_4_ photocatalyst showed highest percentage of degradation against Rhodamine B compared to all other dyes. Thus g-C_3_N_4_ offer advantages as effortless and fast electron transfer extended π-conjugated system and good photocatalytic activity.

## 2 Experimental

### 2.1 Chemicals required

Urea [(NH_2_)_2_CO], Methylene Blue (C_16_H_18_ClN_3_S), Rhodamine B (C_28_H_31_ClN_2_O_3_), Crystal Violet (C_25_N_3_H_30_Cl), Methyl Orange (C_14_H_14_N_3_NaO_3_S), Naphthol Orange (C_16_H_11_N_2_NaO_4_S), *p*-Nitrophenol (C_6_H_5_NO_3_), Terephthalic acid (C_8_H_6_O_4_), Sodium azide (NaN_3_), EDTA (C_10_H_16_N_2_O_8_), Methanol (CH_3_OH), Hydrogen peroxide (H_2_O_2_) were used for this work which were purchased from Merck. The employed chemicals were of analytical grade and used as received without further purification.

### 2.2 Synthesis of g-C_3_N_4_ nanosheets

The synthetic procedure of pure 2D layered g-C_3_N_4_ is shown in Scheme 1. The Urea as precursor was gone through simple one step thermal polycondensation method under a designed temperature in a muffle furnace for the synthesis of g-C_3_N_4_. In particular, 10 g of urea was taken in a pot, finely grounded and then heated on a hotplate for about 10 min which results in colour change from colourless to orange. The resulting solid was transferred to a ceramic crucible with lid and kept in muffle furnace for 3 h with constant heating at 550 °C. The colour of the sample changes from orange to yellow. The yellow product was cooled at room temperature and further grounded in a mortar to obtain fine powder. Finely powdered sample was collected over an air-tight small container and labelled as g-C_3_N_4_ for further uses.

### 2.3 Characterization

Morphologies of the sample were characterized by using scanning electron microscopy and elemental composition of materials was assessed by Energy Dispersive X-ray analysis. SEM image and EDS was recorded on SEM, JEOL model JSM-5800 instrument. The crystallinity and structure of solid samples were profoundly evaluated by X-ray diffraction technique. X-ray diffraction was performed on XPERT-PRO PW3071 diffractometer with Cu Kα (λ = 1.5418 Å) using 40 kV accelerating voltage and 30 mA emission current. Attenuated total reflection infrared spectroscopy provides information related to the presence or absence of functional groups, as well as the chemical structure of polymer materials. ATR-IR was performed using SHIMADZU IR-Affinity-1 FTIR instrument in diamond ATR mode. The surface functional groups and chemical bonding status of the sample was assessed by Fourier transform infrared spectroscopy, recorded on PerkinElmer Paragon 1000 FTIR spectrometer (JEOL JMS-5800). The optical absorption properties of the sample were obtained by a UV–Vis spectrophotometer (PerkinElmer Lambda 35) and the absorbance of the dyes were also recorded by spectrophotometer. Emission spectra of the sample was obtained from fluorescence studies, recorded on JASCO FP-8500 equipped with 150 W Xe source with different excitation wavelength from 200 nm to 700 nm.

### 2.4 Photocatalytic experiment

Here, all the visible light responsive dye degradation studies were recorded by using a 400 W sodium vapour lamp as a source of visible light. The photocatalytic experiment was proceeding through several steps. Firstly, the dye solution was prepared by dissolving the sample in required amount of deionised water. In this study, 0.5 mg/mL of the catalyst and 50 mL of 10^−5^ (M) dye solution was mixed to prepare a suspension, the mixture was sonicated for 10 min and stirred for about 1 h in the dark for attaining adsorption-desorption equilibrium of the dye on the surface of the catalyst. Then, the reaction mixture was put into the quartz tube and visible light was irradiated at different time interval with the help of a spectrophotometer. By drawing aliquot parts of the dye solution at orderly time interval and the progress of the reaction was monitored. The reaction mixture was gone through centrifuged to set apart the catalyst, and the absorbance of the solution was checked. The presence of the residual dye in the supernatant solution is indicated by the intensity of the absorbance maxima. Based on the absorbance, the estimation of dye degradation can be done.

### 2.5 Terephthalic acid test for the formed hydroxyl radicals (^.^OH)

The generation of hydroxyl radicals (^.^OH) in the degradation process was confirmed via Terephthalic acid test. The ^.^OH radicals formed due to the decomposition of H_2_O_2_ in presence g-C_3_N_4_ photocatalyst to produce a fluorescence signal in contact with the Terephthalic acid. The analysis of fluorescence spectra, we use different excitation wavelength such as 310 nm, 315 nm 320 nm and 330 nm respectively. The fluorescence intensity for the conversion of TA to 2-hydroxy TA was measured by the following the λ_ex_/λ_em_ at 315/440 nm.

## 3 Result and discussion

### 3.1 Structural and morphological analysis of g-C_3_N_4_ photocatalyst

**Fig. 1:**
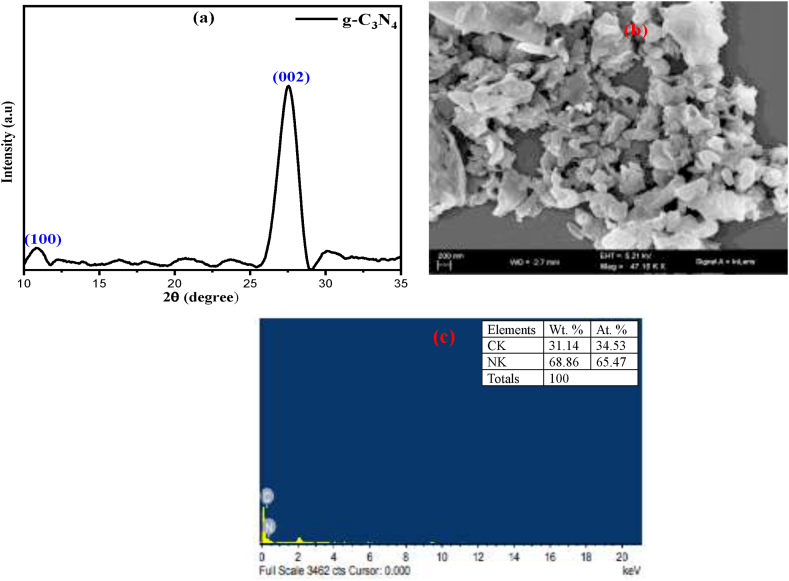
(a) XRD pattern of g-C_3_N_4_ (b) SEM image of g-C_3_N_4_ and (c) EDX of g-C_3_N_4_ (in set elemental composition).

Fig. 1(a) represents the XRD patterns of the synthesized graphitic carbon nitride (g-C_3_N_4_). XRD technique is employed to evaluate the crystal phase of the prepared sample. In Fig. 1(a), it is clearly seen that there are one pronounced diffraction peak 27.5° which was associated with d-spacing = 0.323 nm. The strong peak at 27.5° is likely to be attributed to the long range interplanar stacking of aromatic system, indexed as (002) plane of g-C_3_N_4_ (JCPDS 87–1526) [35]. The small peak at 12.9° corresponds to the periodic arrangement of the triazine units which is labelled as (100) plane with d-spacing = 0.685 nm. It can be said that g-C_3_N_4_ was synthesized with success. The morphology of the g-C_3_N_4_ photocatalyst is observed by SEM as shown in Fig. 1(b). This shows that it is a sheet-like morphology indicating the successfully synthesize the polymeric 2D-graphitic stacking layer structure. Fig. 1(c) shows the EDX spectra of the synthesized g-C_3_N_4_. The image shows the location and the corresponding spectra peak of Carbon(C), and Nitrogen (N) confirmed the presence of g-C_3_N_4_. The corresponding weight and atomic percentage of Carbon(C), and Nitrogen are shown insight Fig. 1(c)

### 3.2 ATR-IR and FTIR analysis

The chemical bonding status and surface functional group analysis for g-C_3_N_4_ was assessed by FTIR technique and the spectra in the range of 500–4000 cm^−1^ was shown in Fig. 1(a) and (b). A sharp band at 807 cm^−1^ is assigned to the breathing mode of the triazine units. The bands at 1617, 1570, and 1410 cm^−1^ is attributed to the C–N stretching vibration of the conjugated aromatic system which eventually proved that g-C_3_N_4_ was successfully synthesized [[36], [37], [38], [39], [40]]. The bands appeared at 1319 and 1248 cm^−1^ were assigned to the stretching vibration of the triazine derivatives like units of C–NH–C or C–N(-C)-C [[37], [38], [39], [40], [41]]. The broad band at 3340 cm^−1^ corresponds to the O–H group of water molecules present on the surface of g-C_3_N_4_ [42].

### 3.3 Raman spectra analysis

**Fig. 2:**
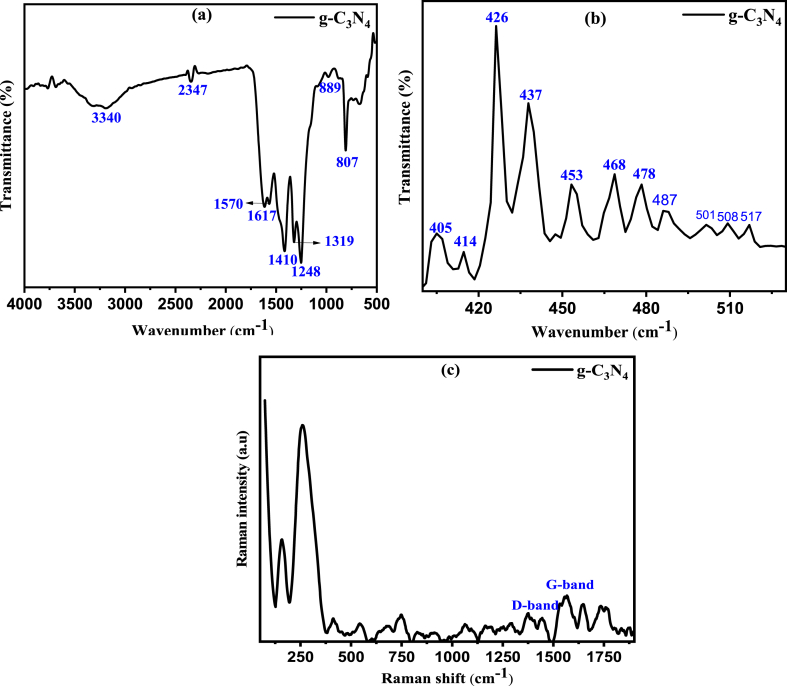
(a) FTIR spectra (b) ATR-IR and (c) Raman spectra of the g-C_3_N_4_ polymeric catalyst.

Fig. 2(c) represents the Raman spectra of the synthesized g-C_3_N_4_ polymer which show almost similar Raman spectra as that of graphite. Raman spectra were recorded for the study of relative intensity between D bands and G bands of the prepared sample which could contemplate the structural impairments of the g-C_3_N_4_. The D bands settled as about 1376 cm^−1^ and are assigned to the structural defects and partly distorted structures of C-sp^2^ while the G bands situated at 1567 cm^−1^, assessed to the degree of graphitization of g-C_3_N_4._ [[43], [44], [45]]. Higher value of I_D_/I_G_ will reveal more structural distortion [43]. The value of I_D_/I_G_ is 0.91 for g-C_3_N_4_, which revealed that there is high structural distortion present in the polymer.

### 3.4 Optical property

**Fig. 3:**
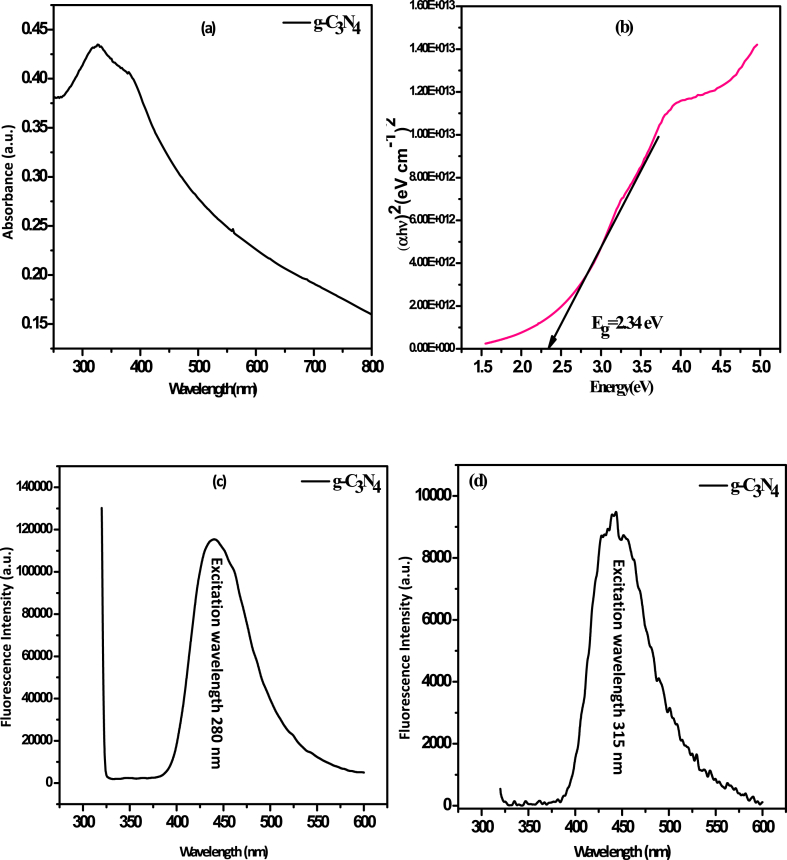
(a) UV–vis spectra of g-C_3_N_4_ (b) Tauc plot of g-C_3_N_4_ photocatalyst having energy bandgap 2.34 eV (c) and (d) the fluorescence spectra of g-C_3_N_4_ excited with two different excited wavelengths 280 nm and 315 nm respectively.

The light absorption property is the key reference for the evaluation of optical property of the synthesized sample. In Fig. 3(a), it is seen that the light absorption band for g-C_3_N_4_ is at 470 nm, this indicates that the g-C_3_N_4_ has visible light absorption ability. The absorption edge is red shifted for pure g-C_3_N_4_. The band gap energy (E_g_) of g-C_3_N_4_ photocatalyst was calculated by Tauc Plot by extrapolating the maximum slope of the plot to the photon energy axes which is shown in Fig. 3(b). The band gap energy (E_g_) can be calculated by using the given equation:

$$\alpha h\vartheta =A{\left(h\vartheta -E_{g}\right)}^{1/2}$$

Where α, h, *ν*, A, and E_g_ are absorption coefficient, plank constant, light frequency, proportionality constant, and band gap energy respectively. The E_g_ value of g-C_3_N_4_ is 2.34 eV as calculated from the formula. As compared to other typical photocatalyst (such as TiO_2_, SnO_2_, ZnO) the g-C_3_N_4_ photocatalyst has smaller band gap energy which justifies the enhanced light absorption of g-C_3_N_4_. The low band gap can be assigned to several intermediate defects present in g-C_3_N_4_ photocatalyst such as uncondensed –NH/-NH_2_ and might have some structural defects such as nitrogen vacancies [46,47].

### 3.5 Fluorescence spectra analysis

Fig. 3(c) and (d) represents the fluorescence emission spectra of g-C_3_N_4_ respectively. The fluorescence spectra of g-C_3_N_4_ shown in Fig. 3(c) has a strong absorption at _∼_319 nm in UV region and an intense peak appeared at 440 nm when excited at 315 nm wavelength arises from the band gap excitation of the as synthesized sample accompanying with the π-π* transitions owing to the triazine structure of g-C_3_N_4_ [48]. The intense peak in the visible range showed the shift of absorption band which comes in support of the n-π* transitions corresponded to the presence of lone pairs on Nitrogen atom of the g-C_3_N_4_ [49]. Generally, the lower the PL signals the higher will be the photoinduced carrier separation efficiency [50]. The signal at 440 nm signifies the enhanced photoinduced carrier separation efficiency and the recombination rate of the light excited electron-hole pair was retarded at smaller extent of the g-C_3_N_4_ photocatalyst [48].

### 3.6 Photocatalytic activity

**Fig. 4:**
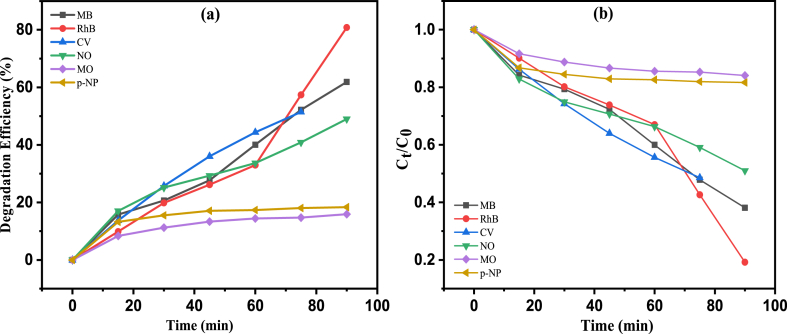
(a) Degradation efficiency of all the selected dyes and (b) Concentration change of all the selected dyes with time in presence of g-C_3_N_4_.

For the investigation of photocatalytic activity of the synthesized g-C_3_N_4_, an experiment regarding photodegradation was performed. From the literature studied of several degradation of organic pollutants by nanocomposites were shown in Table 1, we have studied the degradation of several organic dye pollutants such as Crystal Violet (CV), Methylene Blue (MB), Methyl Orange (MO), Naphthol Orange (NO), and Rhodamine B (RhB). Among the selected dyes, only RhB dye degradation by the g-C_3_N_4_ photocatalyst is effective and more profound. Also we have checked the degradation performance of g-C_3_N_4_ photocatalyst against a phenol derivative (*p*-Nitrophenol). Photocatalysis of g-C_3_N_4_ was checked by using all the selected dyes and the phenol derivative separately and the percentage degradation was noted for all the dyes in order to evaluate the photocatalytic activity of the catalyst in each case. 0.5 mg of the catalyst was mixed with 50 mL of the aqueous solution of each dyes and the phenol derivatives separately and each of the mixtures were stirred for about 1 h in dark to obtain an adsorption-desorption equilibrium, then the mixtures were exposed to sunlight. The maximum absorption (*λ*_max_) of the selected dyes was checked using 3 mL volume of the dyes and the phenol derivative separately using UV–Visible spectrophotometer. The *λ*_max_ values for CV, MB, MO, NO, RhB, and *p*-Nitrophenol are 588, 660, 462, 488, 551, and 400 nm respectively. During the degradation process, a regular time interval was maintained for checking the degradation efficiency with respect to time. Fig. 4 showed that absorption maxima decreases with time in presence of g-C_3_N_4_ photocatalyst and therefore the concentration of the each selected dyes also decrease with time interval. The decolouration and dye degradation efficiency can be calculated by the following formula:

(1) $$D\text{egradation}\;\text{Efficiency}=\frac{\left(A_{o}-A_{t}\right)}{A_{o}}\times 100$$

Where, A_o_ and A_t_ represents the initial absorbance of dyes and absorbance of dyes at time 't' respectively. The percentage of degradation for CV, MB, MO, NO, RhB, and p-NP are 51.44, 61.87, 15.90, 49.00, 80.78, and 18.35 respectively at 90 min.

**Table 1:** A comparison of photocatalytic activity of different catalysts under visible light irradiation.

Photocatalyst	Light source	Application	% Degradation (min)	Reference
g-C_3_N_4_	Visible light	RhB, MB, CV, NO, MO and 4-NP	80.79 %, 61.87 %, 51.44 %, 49.0 %, 15.9 %, 18.37 % in 90 min	This Work
TiO_2_@g-C3N4	Visible light	MB, RhB	95 % in 150min, 96 % in 120 min	[53]
1 % Ag/WO_3_	Visible light	MB dye	80 % in 120min	[54]
Ag NPs	Visible light	Congo red	92 % in 180min	[55]
GO/ZnO	Visible light	MB dye	84 % in 90min	[56]
ZrO_2_/g-C_3_N_4_	Visible light	4-nitrophenol	95 % in 120min	[57]
Bt-OA-CN	Visible light	bisphenol A	Completed in 150 min	[58]
CN/ZnTi-MMO	Visible light	ciprofloxacin	Completed in 20min	[59]
g-C_3_N_4_/Fe_3_O_4_/RGO	Visible light	RhB	100 % in 75min	[60]
sulfuric acid-treated g-C_3_N_4_ (SCN)	Visible light	MB	100 % in 90min	[61]

**Fig. 5:**
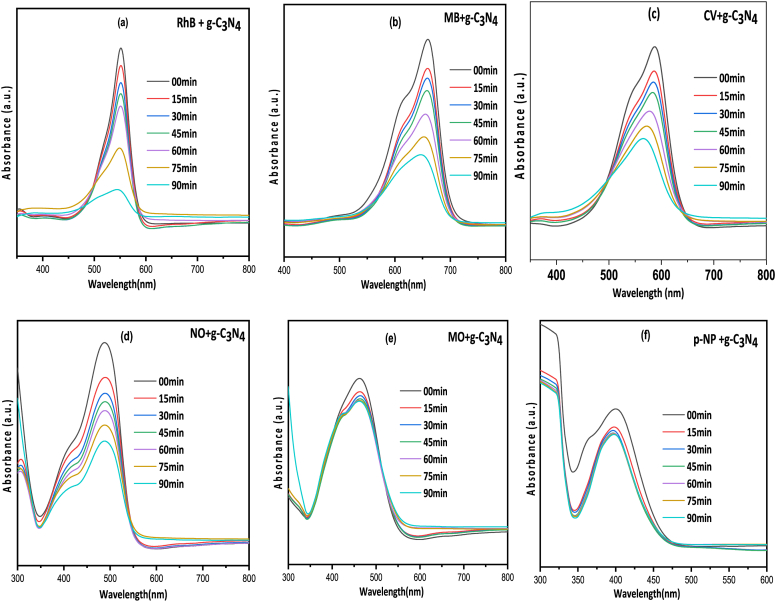
UV–Visible spectra for the concentration changes of different dyes with time (a) RhB (b) MB (c) CV (d) NO (e) MO (f) p-NP.

From Fig. 5(a–f), it is obvious that RhB dyedegraded at faster rate by the photocatalyst with _∼_81 % degradation efficiency and also MB and CV shows high degradation rate but lower in comparison to RhB dye degradation rate. The degradation rate for NO is pretty much appreciable while MO shows least degradation rate among the selected dyes. Fig. 5(f) represents the rate of degradation of p-NP by g-C_3_N_4_ catalyst but the percentage degradation is only 18.35. The g-C_3_N_4_ catalyst has high surface area and small band gap (2.33 eV) and due to these properties it is degraded almost all the selected dyes at greater extent. Degradation of RhB is much higher by the catalyst and this result can be explained by high adherence of the dye on the surface of the catalyst which favours the degradation process [51].

### 3.7 Kinetic study

Detailed kinetic study of the as prepared g-C_3_N_4_was done by using several kinetic models such as zero order kinetic model, first order kinetic model, second order kinetic model, and Behnajady, Modirshahla and Ghanbery (BMG) kinetic model and the expressions of these models are as follows [52]:

(2) $$c_{0}-c_{t}=k_{0}t$$

(3) $$\ln\left(\frac{C_{t}}{C_{0}}\right)=-k_{1}t$$

(4) $$\frac{1}{C_{t}}-\frac{1}{C_{0}}=k_{2}t$$

(5) $$\frac{t}{\left(1-\frac{C_{t}}{C_{0}}\right)}=m+bt$$

Where, c_0_(mg/L), c_t_(mg/L) denoted to the initial concentration of dye and the concentration of the dye at time 't' respectively. K_0_(min), k_1_(min^−1^), k_2_(mg^−1^Lmin^−1^) are the rate constants of zero order, first order, and second order kinetic model respectively and m is a constant (min^−1^) while b is the dimensionless constant.

**Fig. 6:**
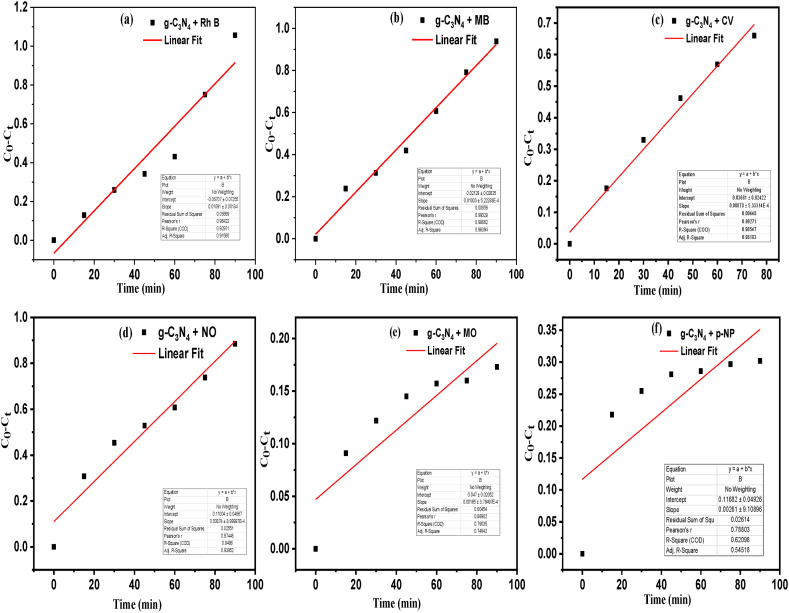
Kinetic curves of different Dyes degradations under same reaction conditions follows Zero order kinetics (a) RhB (b) MB (c) CV (d) NO (e) MO (f) p-NP.

Table 2 depicts the rate constants and R^2^ values of the selected dyes for different kinetic models. With accordance to the linear fitting of the different kinetic models for the degradation of selected dyes, degradation process fits to zero order kinetic model for both MB dye (R^2^ = 0.983) and RhB dye (R^2^ = 0.915), first order kinetic model for CV (R^2^ = 0.999) and NO dye (R^2^ = 0.966), BMG model for both MO (R^2^ = 0.967) and p-NP (R^2^ = 0.993) respectively. Fig. 6(a)-(f) are predicted the rate constant values with respect to the fitted kinetic models and the rate constants are 1 × 10^−2^, 1.1 × 10^−2^, 9.6 × 10^−3^, 6.7 × 10^−3^, 1.7 × 10^−2^, and 4.6 × 10^−2^ for MB, RhB, CV, NO, MO, and p-NP respectively.

**Table 2:** Kinetic parameters of all the selected dyes obtained from different kinetic models under the same reaction conditions.

Dye	Zero order k_o_(min)	R^2^	First order k_1_(min^−1^)	R^2^	Second order k_2_(mg^−1^Lsec^−1^)	R^2^	BMG model 1/m (min^−1^)	1/b	R^2^
MB	1 × 10^−2^	0.983	1 × 10^−2^	0.953	1.1 × 10^−2^	0.883	1.5 × 10^−2^	0.780	0.434
RhB	1.1 × 10^−2^	0.915	1.5 × 10^−2^	0.751	2.7 × 10^−2^	0.551	1.0 × 10^−2^	1.303	−0.003
CV	8.7 × 10^−3^	0.982	9.6 × 10^−3^	0.999	1.1 × 10^−2^	0.990	2.1 × 10^−2^	0.644	0.587
NO	8.7 × 10^−3^	0.939	6.7 × 10^−3^	0.966	5.3 × 10^−3^	0.959	2.2 × 10^−2^	0.526	0.803
MO	1.6 × 10^−3^	0.748	1.6 × 10^−3^	0.770	1.7 × 10^−3^	0.791	1.7 × 10^−2^	0.167	0.967
P-NP	2.6 × 10^−3^	0.545	1.7 × 10^−3^	0.569	1.2 × 10^−3^	0.592	4.6 × 10^−2^	0.188	0.993

### 3.8 Effect of H_2_O_2_ and peroxodisulphate (PDS) on degradation of RhB dye

**Fig. 7:**
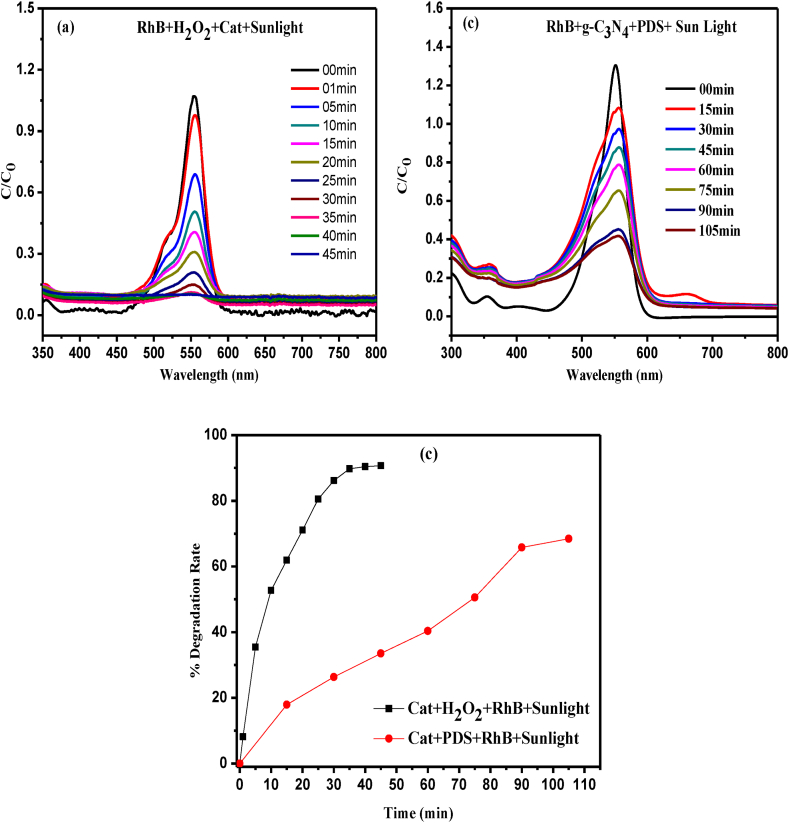
(a) Effect of PDS on degradation of RhB in presence of g-C_3_N_4_ (b) Effect of PDS on degradation of RhB in presence of g-C_3_N_4_ and (c) % degradation rate of RhB in presence of both PDS and H_2_O_2_ respectively.

For the degradation of Rh B dye we have further studied the degradation of organic pollutant in presence of H_2_O_2_ and PDS respectively. For this purpose here we have taken Rh B dye as a model molecule to carry out the reaction. The rate of degradation changes effectively by the catalyst. It was found that the concentration of Rh B decreased by the catalyst in presence of both H_2_O_2_ and PDS respectively. The g-C_3_N_4_ degrades the model dye ∼91 % in presence of H_2_O_2_ at 45 min shown in Fig. 7(a). But in presence of PDS degradation was only ∼68 % at 105 min shown in Fig. 7(b). These results indicated that the rate of degradation is strongly influenced by **.**OH radicals generated by the catalyst in the precursor solution of the reaction instead of the generation of sulphate ion radical from PDS in the degradation process which was not effective catalyst for the process. The percentage degradation rate of Rh B dye in presence of both H_2_O_2_ and PDS is shown in Fig. 7(c).

### 3.9 Scavenging effect and ^.^OH radicals detection

**Fig. 8:**
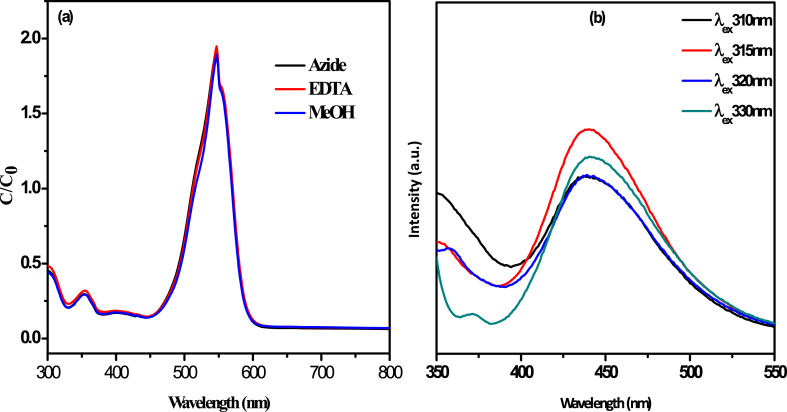
(a) Scavenging effect of NaN_3_, EDTA and MeOH on degradation of RhB in presence of g-C_3_N_4_ (b) detection of ^.^OH radicals by fluorescence spectra.

In the photodegradation of organic dyes in water, it is well known that the reactive species such as singlet oxygen (^1^O_2_), superoxide anion radical (^.^O_2_^−^), hydroxyl radical (^.^OH) and positive hole (h^+^) play important roles in photocatalytic process [62]. In order to better understand whether these species are involved in the degradation process using g-C_3_N_4_ nanosheet, we conducted a series of control experiments (Fig. 8(a)) using g-C_3_N_4_ as catalyst with the following scavengers: Sodium azide as ^1^O_2_ scavenger, Benzoquinone as ^.^O_2_^−^ scavenger, Isopropanol as ^.^OH scavenger and ethylenediaminetetraacetic acid (EDTA) as h^+^ scavenger. In Fig. 8(a) shown that the effect of different scavengers like sodium azide (NaN_3_), EDTA and MeOH were used to measure the degradation of RhB in presence of g-C_3_N_4_. It was found that the concentration of RhB was remained same after 90 min of the reaction shown in Fig. 8(a). This indicated the response of the catalyst for dye degradation was strongly prevented in the presence of different scavenging species. These results suggested that the generation of different oxygen reactive species is quenched by the scavengers effectively. Fig. 8(b) revealed the presence of **.**OH radicals, one of the effective species in the degradation process, studied by fluorescence spectra. In this experiment we have taken TA solution with the addition of 2 mL 30 % H_2_O_2_ in presence of g-C_3_N_4_ photocatalyst. The spectra were recorded at different excitation wavelength (310 nm, 315 nm, 320 nm and 330 nm respectively) and the emission spectrum signal was found at 440 nm. This result suggested the strong existence of.OH radicals generated in presence of g-C_3_N_4_. The intense emission peak was appeared at 440 nm with the excitation wavelength of 315 nm shown in Fig. 8(b).

### 3.10 Photo stability of g-C_3_N_4_ nanocatalyst

**Fig. 9:**
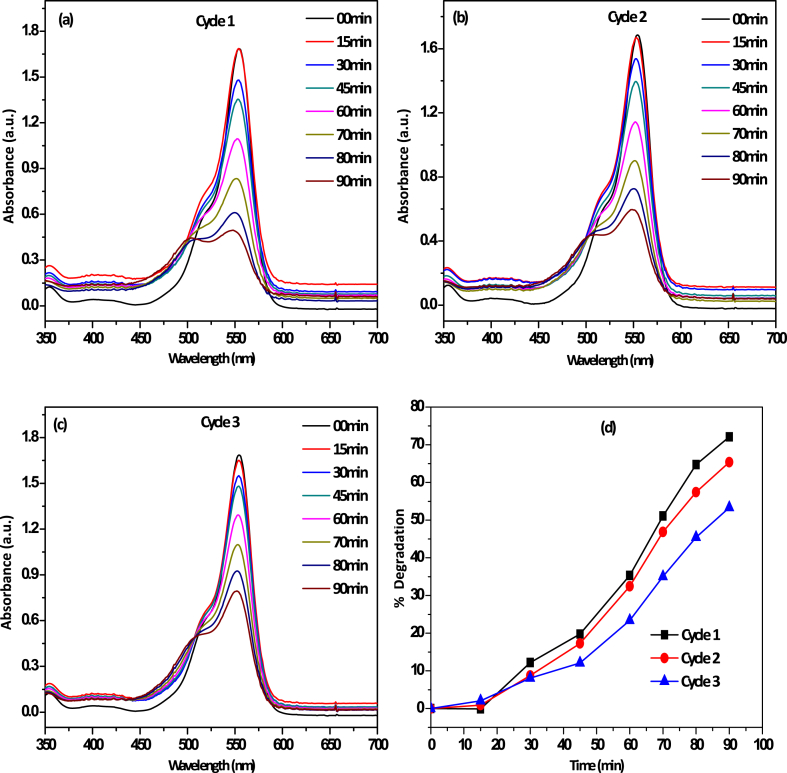
(a) UV–Vis spectra of Rh B degradtion over g-C_3_N_4_ nanosheets under the optimal conditions after 1st cycle (b) UV–Vis spectra of Rh B degradtion over g-C_3_N_4_ nanosheets under the optimal conditions after 2nd cycle (c) UV–Vis spectra of Rh B degradtion over g-C_3_N_4_ nanosheets under the optimal conditions after 3rd cycle (d) Percentage degradation with time interval after each catalytic cycle.

To verify the sustainable application for the removal of dyes, we carried out the three catalytic cycle process shown in Fig. 9(a)-(c). In the degradation process, the residual Rh B concentration was determined in particular time interval and the catalyst was collected at the end of each cycle by filtration and dried before using it in the next cycle. During the three cycle reaction process, the Rh B degradation rate slightly decreased in each cycle and degradation rate at the 3rd cycle was about 53 % at 90 min (Fig. 9(c)). This is probably due to g-C_3_N_4_ leaching during the reaction process. We also dried the catalyst only at 150 °C in a hot air oven instead of calcined at 450 °C as did for the initial g-C_3_N_4_ catalyst.

## 4 Degradation mechanism

The band edge potential of any semiconducting material is the key factor to determine the transfer of photo generated charges to the targeting substances. Therefore the band edge potential of g-C_3_N_4_ sample was computed using the following equations [63]:

(6) E_VB_ = χ+ 0.5E_g_-E^e^

(7) E_CB_ = E_VB_-E_g_

**Fig. 10:**
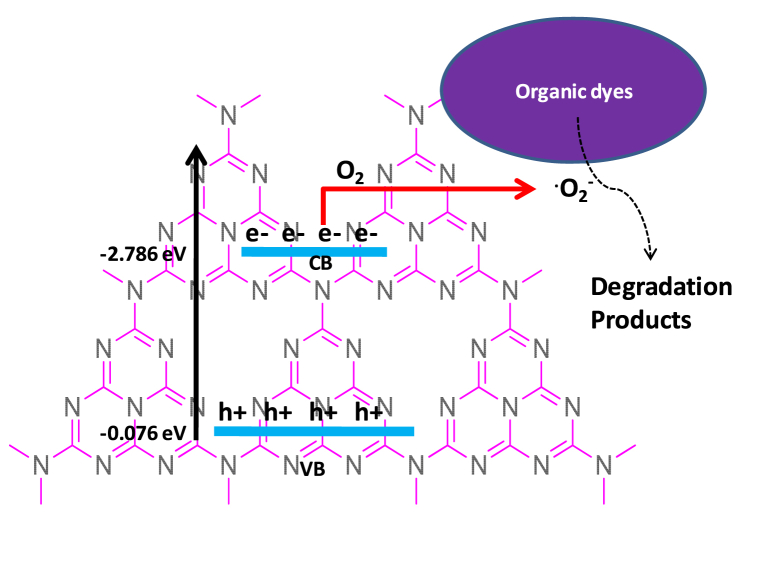
Mechanistic diagram of g-C_3_N_4_ and photo-degradation of organic dyes under visible light.

Here, χ, E_g_, E_VB_, and E_CB_, denotes to the electronegativity, band gap, valence band edge potential, and conduction band edge potential of the semiconducting material, respectively whereas, E_e_ is the free electron energy (eV) relative to the hydrogen-scale (∼4.25 eV) [60]. Electronegativity of g-C_3_N_4_ is calculated by using the given equation:

(8) χ = [χ(A)^a^ χ(B)^b^ ]^1/a+b^

where a, b are the number of atoms in the g-C_3_N_4_, while the electronegativity of carbon and nitrogen was calculated as the arithmetic mean of the atomic electron affinity and the first ionization energy. The value of χ of g-C_3_N_4_ was calculated to be 2.819 while E_CB_ and E_VB_ were estimated to be −2.786 eV and −0.076 eV respectively shown in Fig. 10. The VB potential of g-C_3_N_4_ (−0.076 eV) is lower than the redox potential of H_2_O/^.^OH (+2.38 V vs. NHE) and –OH/^**.**^OH (+1.99 eV vs. NHE). The results indicate the generation of ^.^OH radicals by the reaction with VB photo-holes of g-C_3_N_4_ and H_2_O/-OH which are the reactive species in the photo-degradation process.

In photo catalytic degradation of organic pollutants, on the exposure to the sunlight the electron in the valence bands are excited to higher energy bands (CB) with formation of electron-hole pairs. An oxygen reactive species (ROS) in the degradation process originates from such electron-hole pairs and the ROS attacks the organic dyes and convert the dyes into small fragments.

(9) g-C_3_N_4_ + hν → h^+^ +e^−^

(10) h^+^ +H_2_O → H^+^+ ^**.**^OH

(11) ^**.**^OH + Dye → Degradation Products

(12) O_2_ + e^−^ → ^**.**^O_2_^−^

(13) ^**.**^O_2_^−^ + Dye → Degradation Products

## 5 Conclusion

In summary, we have reported a simple method to realize an optimized 2D layer g-C_3_N_4_ using sealed thermal condensation process. The highly porous morphology, favorable electronic properties such as band gap and PL studies showed that g-C_3_N_4_ facilitating a better adsorption of the dye on it and visible light induced photocatalytic activity with appreciable improvement in the performance. The optical properties and internal structure of the 2D layered g-C_3_N_4_ have been analyzed by the use of both FTIR and Raman spectroscopy. The PL study in the 468 nm region indicating the optical band gap matched with the calculated value obtained from UV–visible studies. The calculated band gap of g-C_3_N_4_ was found to be 2.34 eV. The photocatalytic degradation of well-known organic pollutants such as MB, RhB, CV, NO, MO, and p-NP respectively was evaluated by the used of layered g-C_3_N_4_ under visible light irradiation. Among them RhB showed highest degradation rate in the process under same condition of reaction. The Scavenger experiment suggests that the photocatalytic degradation mechanism involves the electrons as the majority charge carriers along with other reactive species in the degradation process. We can hopefully say that our work will be beneficial in the photocatalytic reactions in future.

## Data availability

The data that support the findings of this work are available from the corresponding author upon reasonable request.

## CRediT authorship contribution statement

**Afroja Banu:** Writing – review & editing, Writing – original draft, Methodology, Investigation, Formal analysis, Data curation. **Biswajit Sinha:** Writing – review & editing, Writing – original draft, Supervision, Conceptualization. **Suranjan Sikdar:** Writing – review & editing, Writing – original draft, Supervision, Conceptualization.

## Declaration of Competing interest

The authors declare the following financial interests/personal relationships which may be considered as potential competing interests: Dr. Suranjan Sikdar reports administrative support was provided by University of North Bengal. Afroja Banu reports a relationship with University of North Bengal that includes: non-financial support. N A has patent NA pending to NA. Not Applicable If there are other authors, they declare that they have no known competing financial interests or personal relationships that could have appeared to influence the work reported in this paper.

## References

[1] Prasad C., Tang H., Bahadur I. (2019). Graphitic carbon nitride based ternary nanocomposites: from synthesis to their applications in photocatalysis: a recent review. J. Mol. Liq..

[2] Ahmed S., Rasul M.G., Martens W.N., Brown R., Hashib M.A. (2010). Heterogeneous photocatalytic degradation of phenols in wastewater: a review on current status and developments. Desalination.

[3] Mu’azu N., Jarrah N., Zubair M., Alagha O. (2017). Removal of phenolic compounds from water using Sewage Sludge-based activated carbon adsorption: a review. Int. J. Environ. Res. Publ. Health.

[4] Mohamad Said K.A., Ismail A.F., Abdul Karim Z., Abdullah M.S., Hafeez A. (2021). A review of technologies for the phenolic compounds recovery and phenol removal from wastewater. Process Saf. Environ. Protect..

[5] Singh A.K., Bilal M., Iqbal H.M.N., Meyer A.S., Raj A. (2021). Bioremediation of lignin derivatives and phenolics in wastewater with lignin modifying enzymes: status, opportunities and challenges. Sci. Total Environ..

[6] O'Shea K.E., Dionysiou D.D. (2012). Advanced oxidation processes for water treatment. J. Phys. Chem. Lett..

[7] Chen S., Takata T., Domen K. (2017). Particulate photocatalysts for overall water splitting. Nat. Rev. Mater..

[8] Seo J., Lee H., Lee H.-J., Kim M.S., Hong S.W., Lee J., Lee C. (2018). Visible light-photosensitized oxidation of organic pollutants using amorphous peroxo-titania. Appl. Catal. B Environ..

[9] He W., Kim H.-K., Wamer W.G., Melka D., Callahan J.H., Yin J.-J. (2013). Photogenerated charge carriers and reactive oxygen species in ZnO/Au hybrid nanostructures with enhanced photocatalytic and antibacterial activity. J. Am. Chem. Soc..

[10] Hayyan M., Hashim M.A., AlNashef I.M. (2016). Superoxide ion: generation and chemical implications. Chem. Rev..

[11] Feng H., Du Y., Wang C., Hao W. (2017). Efficient visible-light photocatalysts by constructing dispersive energy band with anisotropic p and s - p hybridization states. Curr. Opin. Green Sustainable Chem..

[12] Kumar S.G., Rao K.S.R.K. (2017). Comparison of modification strategies towards enhanced charge carrier separation and photocatalytic degradation activity of metal oxide semiconductors (TiO 2 , WO 3 and ZnO). Appl. Surf. Sci..

[13] Das T.K., Ilaiyaraja P., Mocherla P.S.V., Bhalerao G.M., Sudakar C. (2016). Influence of surface disorder, oxygen defects and bandgap in TiO 2 nanostructures on the photovoltaic properties of dye sensitized solar cells. Sol. Energy Mater. Sol. Cell..

[14] Sopiha K.V., Malyi O.I., Persson C., Wu P. (2021). Chemistry of oxygen ionosorption on SnO2 surfaces. ACS Appl. Mater. Interfaces.

[15] Siriwong C., Wetchakun N., Inceesungvorn B., Channei D., Samerjai T., Phanichphant S. (2012). Doped-metal oxide nanoparticles for use as photocatalysts. Prog. Cryst. Growth Char. Mater..

[16] Xu R., Li J., Sui G., Zhuang Y., Guo D., Luo Z., Lian S., Yao H., Wang C., Chen S. (2022). Constructing supramolecular self-assembled porous g-C_3_N_4_ nanosheets containing thiophene-groups for excellent photocatalytic performance under visible light. Appl. Surf. Sci..

[17] Liang S., Sui G., Guo D., Luo Z., Xu R., Yao H., Li J., Wang C. (2023). g-C_3_N_4_-wrapped nickel doped zinc oxide/carbon core-double shell microspheres for high-performance photocatalytic hydrogen production. J. Colloid Interface Sci..

[18] Ismael M., Wu Y. (2019). A mini-review on the synthesis, structural modification of g-C3N4-based materials, and its applications for solar energy conversion and environmental remediation. Sustain. Energy Fuels.

[19] Darkwah W.K., Ao Y. (2018). Mini review on the structure and properties (photocatalysis), and preparation techniques of graphitic carbon nitride nano-based particle, and its applications. Nanoscale Res. Lett..

[20] Kumar S., Kumar A., Bahuguna A., Sharma V., Krishnan V. (2017). Two-dimensional carbon-based nanocomposites for photocatalytic energy generation and environmental remediation applications. Beilstein J. Nanotechnol..

[21] Sui G., Li J., Du L., Zhuang Y., Zhang Y., Zou Y., Li B. (2020). Preparation and characterization of *g*-C_3_N_4_/Ag–TiO_2_ ternary hollow sphere nano heterojunction catalyst with high visible light photocatalytic performance. J. Alloys Compd..

[22] Das D., Shinde S.L., Nanda K.K. (2016). Temperature-dependent photoluminescence of g-C3N4: implication for temperature sensing. ACS Appl. Mater. Interfaces.

[23] Zhang X., Xie X., Wang H., Zhang J., Pan B., Xie Y. (2012). Enhanced photoresponsive ultrathin graphitic-phase C3N4 nanosheets for bioimaging. J. Am. Chem. Soc..

[24] Tan L., Nie C., Ao Z., Sun H., An T., Wang S. (2021). Novel two-dimensional crystalline carbon nitrides beyond g-C3N4: structure and applications. J. Mater. Chem. A.

[25] Paul D.R., Gautam S., Panchal P., Nehra S.P., Choudhary P., Sharma A. (2020). ZnO-modified g-C3N4: a potential photocatalyst for environmental application. ACS Omega.

[26] Tang J., Xu R., Sui G., G D., Zhao Z., Fu S., Yang X., Li Y., Li J. (2023). Double-shelled porous g-C_3_N_4_ nanotubes modified with amorphous Cu-doped FeOOH nanoclusters as 0D/3D non-homogeneous photo-fenton catalysts for effective removal of organic dyes. Small.

[27] Perveen M., Nazir S., Arshad A.W., Khan M.I., Shamim M., Ayub K., Iqbal J. (2020). Therapeutic potential of graphitic carbon nitride as a drug delivery system for cisplatin (anticancer drug): a DFT approach. Biophys. Chem..

[28] Liao G., He F., Li Q., Zhong L., Zhao R., Che H., Fang B. (2020). Emerging graphitic carbon nitride-based materials for biomedical applications. Prog. Mater. Sci..

[29] Chen H., Tan X., Zhang J., Lu Q., Ou X., Ruo Y., Chen S. (2014). An electrogenerated chemiluminescent biosensor based on a g-C3N4–hemin nanocomposite and hollow gold nanoparticles for the detection of lactate. RSC Adv..

[30] Zhang G., Zhang F., Zhang J., Li J., Jin X., Li Y., Ren W. (2019). Modulating charge transfer dynamics for g-C3N4 through dimension and interface engineered transition metal phosphide co-catalyst for efficient visible-light photocatalytic hydrogen generation. J. Mater. Chem..

[31] Gao X., Yang B., Yao W., Wang Y., Zong R., Wang J., Tao D. (2019). Enhanced photocatalytic activity of ZnO/g-C3N4 composites by regulating stacked thickness of g-C3N4 nanosheets. Environ. Pollut..

[32] Liu L.-L., Chen F., Wu J.-H., Li W.-W., Chen J.-J., Yu H.-Q. (2021). Fine tuning of phosphorus active sites on g-C3N4 nanosheets for enhanced photocatalytic decontamination. J. Mater. Chem. A.

[33] Zhu D., Zhou Q. (2020). Nitrogen doped g-C3N4 with the extremely narrow band gap for excellent photocatalytic activities under visible light. Appl. Catal. B Environ..

[34] Chidhambaram N., Ravichandran K. (2017). Single step transformation of urea into metal-free g-C 3 N 4 nanoflakes for visible light photocatalytic applications. Mater. Lett..

[35] Thomas A., Fischer A., Goettmann F., Antonietti M., Müller J.O., Schlögl R., Carlsson J.M. (2008). J. Mater. Chem.

[36] Chen Q., Hao Y., Song Z., Liu M., Chen D., Zhu B., Chen Z. (2021). Optimization of photocatalytic degradation conditions and toxicity assessment of norfloxacin under visible light by new lamellar structure magnetic ZnO/g-C3N4. Ecotoxicol. Environ. Saf..

[37] Mo Z., She X., Li Y., Liu L., Huang L., Chen Z., Zhang Q., Xu H., Li H. (2015). Synthesis of gC3N4 at diferent temperatures for superior visible/UV photocatalytic performance and photoelectrochemical sensing of MB solution. RSC Adv..

[38] Dong F., Li Y., Wang Z., Ho W.-K. (2015). Enhanced visible light photocatalytic activity and oxidation ability of porous graphenelike g-C3N4 nanosheets via thermal exfoliation. Appl. Surf. Sci..

[39] Kim M., Hwang S., Yu J.-S. (2017). Novel ordered nanoporous graphitic C3N4 as a support for Pt–Ru anode catalyst in direct methanol fuel cell. J Chem Mater.

[40] Zhu B., Xia P., Li Y., Ho W., Yu J. (2017). Fabrication and photocatalytic activity enhanced mechanism of direct Z-scheme g-C3N4/Ag2WO4photocatalyst. Appl. Surf. Sci..

[41] Bala I., Gupta S.P., Kumar S., Singh H., De J., Sharma N., Kailasam K., Pal S.K. (2018). Hydrogen-bond mediated columnar liquid crystalline assemblies of C 3-symmetric heptazine derivatives at ambient temperature. Soft Matter.

[42] Narkbuakaew T., Sujaridworakun P. (2020). Synthesis of tri-S-triazine based g-C_3_N_4_ photocatalyst for cationic rhodamine B degradation under visible light. Top. Catal..

[43] Sheng Z., Shao L., Chen J., Bao W., Wang Fe, Xia X. (2011). ACS Nano.

[44] Zhang Y., Pan Q., Chai G., Liang M., Dong G., Zhang Q., Qiu J. (2013). Sci. Rep..

[45] Ferrari A.C., Robertson J. (2000). Phys. Rev. B.

[46] Zhang G., Zhang J., Zhang M., Wang X. (2012). Polycondensation of thiourea into carbon nitride semiconductors as visible light photocatalysts. J. Mater. Chem..

[47] Liu R., Chen Z., Yao Y., Li Y., Cheema W.A., Wang D., Zhu S. (2020). Recent advancements in g-C3N4-based photocatalysts for photocatalytic CO2 reduction: a mini review. RSC Adv..

[48] Mondal T.K., Saha S.K. (2020). Interesting photoluminescence behaviour in graphitic carbon nitride quantum dots attached PbCrO4 colloidal nanostructures. New J. Chem..

[49] Dong G., Zhang Y., Pan Q., Qiu J. (2014). A fantastic graphitic carbon nitride (g-C3N4) material: electronic structure, photocatalytic and photoelectronic properties. J. Photochem. Photobiol. C Photochem. Rev..

[50] han M.E., Han T.H., Khan M.M., Karim M.R., Cho M.H. (2018). Environmentally sustainable fabrication of Ag@g-C3N4 nanostructures and their multifunctional efficacy as antibacterial agents and photocatalysts. ACS Appl. Nano Mater..

[51] D N., Kondamareddy K.K., Bin H., Lu D., Kumar P., Dwivedi R.K., Fu D. (2018). Enhanced visible light photodegradation activity of RhB/MB from aqueous solution using nanosized novel Fe-Cd co-modified ZnO. Sci. Rep..

[52] Banu A., Barman S., Sinha B., Sikdar S. (2023). Synthesis of tetragonal SnO_2_ photocatalyst for Micro-structural analysis and visible light driven Fenton-like degradation of Methylene Blue. ChemistrySelect.

[53] Madima Ntakadzeni, Kefeni Kebede K., Mishra Shivani B., Mishra Ajay K. (2022). TiO_2_-modified g-C_3_N4 nanocomposite for photocatalytic degradation of organic dyes in aqueous solution. Helion.

[54] Matalkeh Maha, Nasrallah Gheyath K., Shurrab Farah M., Al-Absi Enas S., Mohammed Widad, Ahmed Elzatahry, Saoud Khaled M. (2022). Visible light photocatalytic activity of Ag/WO_3_ nanoparticles and its antibacterial activity under ambient light and in the dark. Results in Engineering.

[55] Thirumagal N., Jeyakumari A.P. (2020). Photocatalytic and antibacterial activities of AgNPs from mesua ferrea seed. SN Appl. Sci..

[56] Al-Rawashdeh N.A.F., Allabadi O., Aljarrah M.T. (2020). Photocatalytic activity of graphene oxide/zinc oxide nanocomposites with embedded metal nanoparticles for the degradation of organic dyes. ACS Omega.

[57] Zarei M., Bahrami J., Zarei M. (2019). Zirconia nanoparticle-modified graphitic carbon nitride nanosheets for effective photocatalytic degradation of 4-nitrophenol in water. Appl. Water Sci..

[58] Katsumata H., Higashi F., Kobayashi Y., Tateishi I., Furukawa M., Kaneco S. (2019). Dual-defect-modified graphitic carbon nitride with boosted photocatalytic activity under visible light. Sci. Rep..

[59] Chuaicham Chitiphon, Sekar Karthikeyan, Balakumar Vellaichamy, Mittraphab Yanisa, Shimizu Kuniyoshi, Ohtani Bunsho, Sasaki Keiko (2022). Fabrication of graphitic carbon nitride/ZnTi-mixed metal oxide heterostructure: robust photocatalytic decomposition of ciprofloxacin. J. Alloys Compd..

[60] Luo J., Dai Z., Feng M. (2023). Graphitic carbon nitride/ferroferric oxide/reduced graphene oxide nanocomposite as highly active visible light photocatalyst. Nano Res..

[61] Pawar R., Kang S., Park J. (2016). Room-temperature synthesis of nanoporous 1D microrods of graphitic carbon nitride (g-C_3_N_4_) with highly enhanced photocatalytic activity and stability. Sci. Rep..

[62] Raja P., Bozzi A., Mansilla H., Kiwi J. (2005). Evidence for superoxide-radical anion, singlet oxygen and OH-radical intervention during the degradation of the lignin model compound (3-methoxy-4-hydroxyphenylmethylcarbinol). J. Photochem. Photobiol. Chem..

[63] Uribe López M.C., Alvarez Lemus M.A., Hidalgo M.C., López González R., Quintana Owen P., Oros-Ruiz S., Acosta J. (2019). J. Nanomater..

